# The Role of Troponin in the Diagnosis and Treatment of Acute Pulmonary Embolism: Mechanisms of Elevation, Prognostic Evaluation, and Clinical Decision-Making

**DOI:** 10.7759/cureus.67922

**Published:** 2024-08-27

**Authors:** Liu Yang, Bin Li, Huaigang Chen, N. Belfeki, M. Monchi, C. Moini

**Affiliations:** 1 Department of Cardiology, Jiangxi Provincial People's Hospital, The First Affiliated Hospital of Nanchang Medical College, Nanchang, CHN; 2 Department of Cardiology, Jiangxi Medical College, Nanchang University, Nanchang, CHN; 3 Department of Internal Medicine, Groupe Hospitalier Sud Île-de-France, Melun, FRA; 4 Intensive Care Unit, Groupe Hospitalier Sud Île-de-France, Melun, FRA; 5 Department of Cardiology, Groupe Hospitalier Sud Île-de-France, Melun, FRA

**Keywords:** mechanisms, prognostic evaluation, diagnosis, troponin, acute pulmonary embolism

## Abstract

Acute pulmonary embolism (APE) is a cardiovascular disease with severe consequences, wherein cardiac troponin (Tn) plays a pivotal role in diagnosis and treatment. This article reviews the various roles of Tn in managing APE. It looks at how Tn levels increase, their importance in predicting outcomes, and their use in making clinical decisions. Studies indicate that an elevation in Tn is primarily associated with right ventricular overload, ischemia, and necrosis, changes that directly reflect the extent of right ventricular dysfunction and myocardial injury. Elevated levels of Tn are significantly correlated with both short-term and long-term mortality risks in patients with APE, serving as crucial indicators for prognostic assessment and guiding therapeutic strategies. International guidelines recommend integrating Tn testing with clinical scoring and echocardiography to optimize treatment decisions in patients with APE. Despite the significant value of Tn determination in the management of APE, further research is needed to standardize its application. This paper emphasizes future research directions, including exploring the application of Tn in different patient subgroups with APE and its potential combined use with other biomarkers.

## Introduction and background

Acute pulmonary embolism (APE) is a severe cardiovascular disorder characterized by obstructing the pulmonary artery or its branches by thrombi or other substances, resulting in pulmonary circulatory impairment and ischemia [1]. The incidence of pulmonary embolism (PE) ranges from 39 to 115 per 100,000 population annually; for DVT, the incidence ranges from 53 to 162 per 100,000 people. APE is a common and perilous disease, characterized by a high incidence and mortality rate [2,3]. Because of the diverse and non-specific clinical manifestations of APE, such as dyspnea, chest pain, and hemoptysis, the diagnosis is often challenging and delayed.

According to guidelines from the European Society of Cardiology and the American College of Chest Physicians, patients with APE can be classified into three categories based on their risk of mortality: high risk, intermediate risk, and low risk [4]. High-risk patients require urgent thrombolysis or surgical treatment, medium-risk patients need close monitoring and anticoagulation therapy, and low-risk patients can be observed in the outpatient or inpatient setting and receive anticoagulation therapy [5]. Therefore, risk stratification of patients with APE and selecting appropriate treatment strategies based on their risk level are crucial in reducing mortality and improving prognosis. Currently, the methods for diagnosing and determining APE and its prognosis mainly include clinical scoring, laboratory testing, and imaging examinations. However, these modalities all have limitations and inadequacies. Therefore, there is an urgent clinical need to identify a sensitive and specific diagnostic biomarker to improve the accuracy and timeliness of APE diagnosis.

Troponin (Tn) is a regulatory protein found in cardiomyocytes [6]. When myocardial cells undergo necrosis, Tn is released into the bloodstream and can be detected through enzyme-linked immunosorbent assays (ELISA) or chemiluminescence methods. Over the decades, an increasing body of research has highlighted the significant clinical relevance of measuring Tn levels in patients with APE [7-9]. It can not only reflect the degree of right ventricular dysfunction and myocardial injury caused by APE but can also serve as an important prognostic biomarker to help assess APE patients' risks of short-term and long-term mortality, right ventricular impairment, and other adverse outcomes.

This article aims to review the role of Tn in patients with APE, including its mechanisms of elevation, role in prognostic assessment, and assistance in clinical decision-making. We will analyze the various causes of elevated Tn, its relationship with the prognosis of patients with APE, and how to guide diagnostics and treatment based on Tn levels. This review also evaluates the current global guidelines on Tn testing and explores its application and limitations in the diagnosis and treatment of APE. Finally, we will highlight the current research gaps and propose directions for future studies.

## Review

 Methods

 Literature Search

* *Retrieval should be conducted using PubMed, EMBASE, and Sino Med databases. Search terms predefined in titles, abstracts, and keywords are used to identify pertinent studies. More information about the terms used in the search can be defined as followings: (Troponin [Title/Abstract]) AND (Pulmonary Embolism [Title/Abstract]), (Troponin [Title/Abstract]) AND (Right Ventricular Dysfunction [Title/Abstract]), (Troponin [Title/Abstract]) AND (Prognosis [Title/Abstract]), (Troponin [Title/Abstract]) AND (Diagnosis [Title/Abstract]), (Troponin [Title/Abstract]) AND (Mortality [Title/Abstract]), (Troponin [Title/Abstract]) AND (Risk Stratification [Title/Abstract]), (Troponin [Title/Abstract]) AND (Biomarker [Title/Abstract]), (Troponin [Title/Abstract]) AND (Myocardial Injury [Title/Abstract]), (Troponin [Title/Abstract]) AND (Acute Pulmonary Embolism [Title/Abstract]), (Troponin [Title/Abstract]) AND (Cardiomyocytes [Title/Abstract]), (Cardiac Biomarkers [Title/Abstract]) AND (Acute Pulmonary Embolism [Title/Abstract]), (Troponin I[Title/Abstract]) AND (Pulmonary Embolism [Title/Abstract]), (Troponin T[Title/Abstract]) AND (Pulmonary Embolism [Title/Abstract]), (High-sensitivity Troponin [Title/Abstract]) AND (Pulmonary Embolism [Title/Abstract]), (Troponin [Title/Abstract]) AND (Short-term Mortality [Title/Abstract]), (Troponin [Title/Abstract]) AND (Long-term Mortality [Title/Abstract]), (Troponin [Title/Abstract]) AND (Echocardiography [Title/Abstract]), (Troponin [Title/Abstract]) AND (CTPA[Title/Abstract]), (Troponin [Title/Abstract]) AND (Thrombolysis [Title/Abstract]), (Troponin [Title/Abstract]) AND (Risk Assessment [Title/Abstract]). The retrieval period spans from the inception of the databases up to November 2023.

Mechanisms of Elevated Tn in Patients With APE

This section will also explore the effects of right ventricular dysfunction associated with molecular mechanisms on Tn levels. Additionally, we will present other potential confounding factors, such as myocarditis or pericarditis, that could indirectly lead to elevated Tn.

Right Ventricular Overload, Ischemia, and Necrosis Caused by APE

When the pulmonary artery or its branches are obstructed by thrombi or other substances, pulmonary circulatory resistance sharply increases, leading to augmented afterload on the right ventricle [10]. If the right ventricle fails to adapt to this acute pressure overload, changes such as right ventricular dilation, wall thinning, reduced contractility, and decreased compliance occur. These alterations affect the coronary perfusion pressure of the right ventricle, leading to right ventricular ischemia. If the ischemia persists or intensifies, it can result in right ventricular necrosis. Right ventricular ischemia and necrosis lead to the release of Tn into the bloodstream.

Therefore, the elevation of Tn in patients with APE is primarily attributable to the right ventricular overloading, ischemia, and necrosis caused by APE itself, and it reflects the severity of right ventricular dysfunction.

Other Potential Factors Leading to Elevated Tn

Apart from right ventricular overload, ischemia, and necrosis caused by APE, several other factors may contribute to an elevation in Tn levels, such as coexisting myocarditis, pericarditis, heart failure, and coronary artery atherosclerosis. These conditions can lead to dysfunction of the left or both ventricles, thereby affecting the pulmonary circulation and the load on the right ventricle, or directly damaging myocardial cells, causing the release of Tn.

However, aside from right ventricular overload, ischemia, necrosis, and other cardiac diseases, there are intriguing molecular mechanisms that might contribute to elevated Tn in patients with APE. One possible mechanism is myocardial cell apoptosis or programmed cell death of myocardial cells. Additionally, when myocardial cells are stressed or damaged, certain signaling pathways are activated, leading to apoptosis of these cells. Apoptotic myocardial cells release cellular contents, including Tn, into the bloodstream, increasing Tn levels.

Other Intriguing Molecular Mechanisms Potentially Elevating Tn in Patients With APE

**Figure 1:**
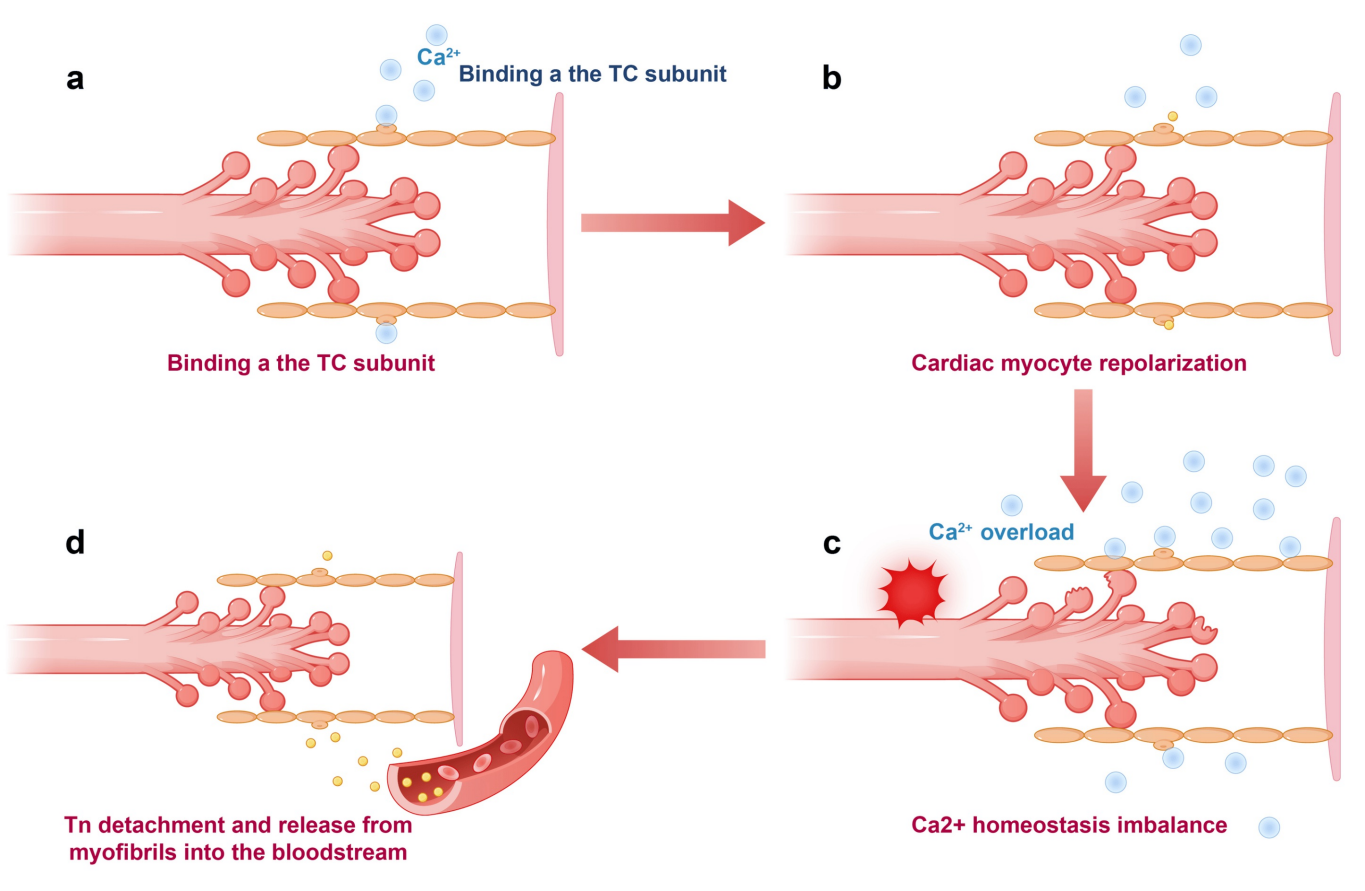
Myocardial cell calcium dynamics and troponin (Tn) release mechanism. (a) When myocardial cells are excited, extracellular Ca2+ enters the cell and triggers the release of more Ca2+ from the sarcoplasmic reticulum, forming an influx and binding to the C subunit of Tn (TnC), leading to the interaction of actin and myosin, generating contractile force. TnC binds to calcium ions, which is crucial for the regulation of muscle contraction. (b) Calcium efflux and myocardial relaxation: During myocardial cell repolarization, Ca2+ is expelled through calcium pumps and exchange proteins, restoring the original position of the Tn I subunit, causing the separation of actin and myosin, and resulting in relaxation force. (c) Myocardial stress and calcium overload: Excessive stress or injury (such as ischemia and hypoxia) to myocardial cells leads to calcium imbalance and overload. (d) Calcium overload and myocardial cell apoptosis: Calcium overload causes mitochondrial dysfunction and inhibition of ATP synthesis, leading to myocardial cell apoptosis. The blood level of Tn reflects the extent of myocardial cell damage and the state of cardiac function. The authors of this article used FigDraw (https://www.figdraw.com/#/paint_about).

Myocardial cell calcium overload: When pulmonary artery resistance suddenly increases, the right ventricle requires greater contractile force to maintain blood flow, which elevates the metabolic demands of myocardial cells and the influx of calcium ions. The accumulation of calcium ions within myocardial cells leads to calcium overload [11], calcium overload is a factor that leads to myocardial cell damage, and the release of Tn into the bloodstream is primarily a direct consequence of this cellular injury [12], as illustrated in Figure 1.

**Figure 2:**
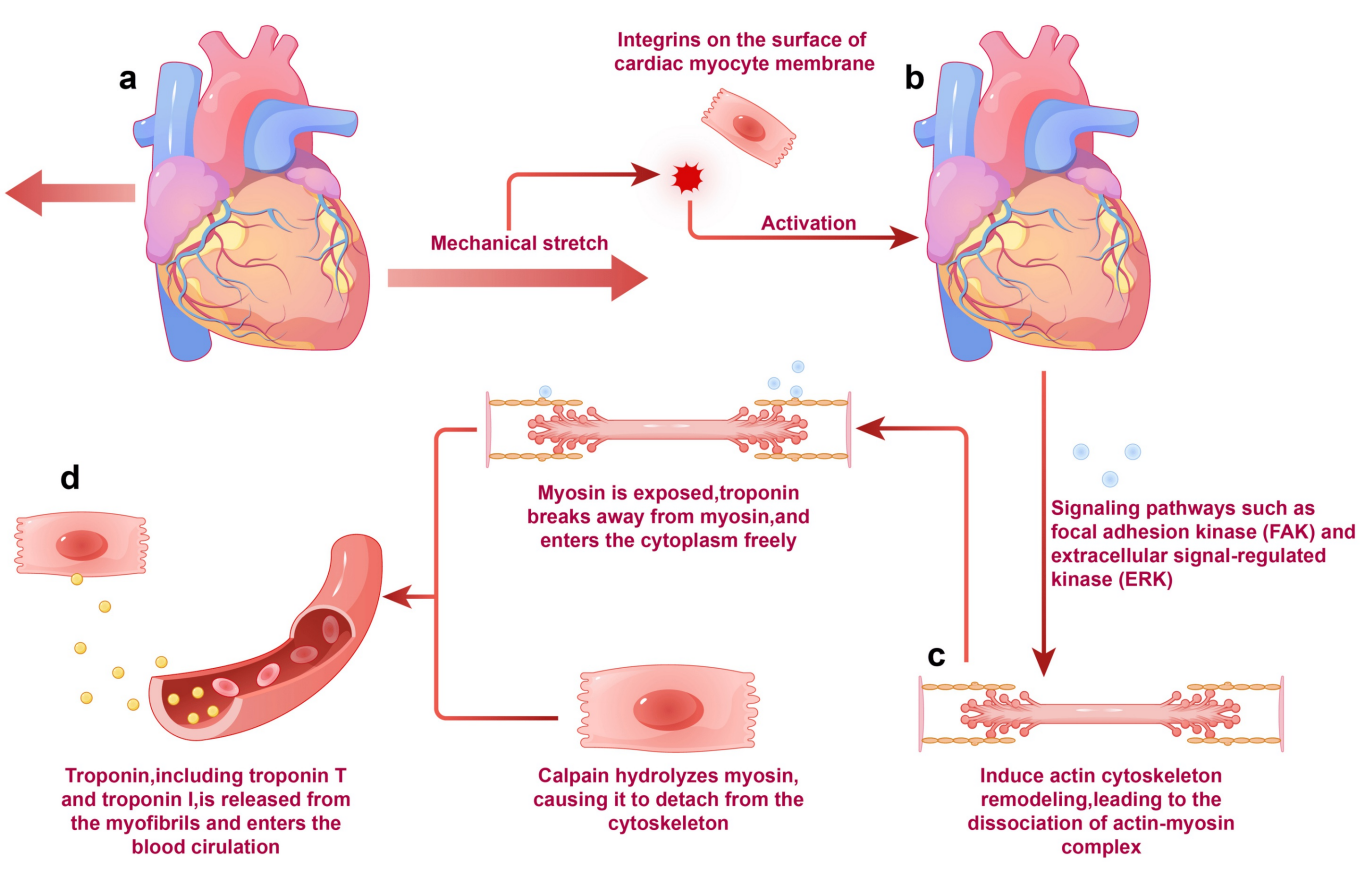
Mechanical stretching and stress response of cardiomyocytes due to right ventricular dilation and their molecular mechanisms. (a) Mechanical stretching and stress response of cardiomyocytes; (b) activation of key signaling pathways within cardiomyocytes, especially integrin-mediated signal transduction pathways; (c) MAPK, JAK/STAT, and calpain-dependent pathways; and (d) release of Tn from muscle fibers. The authors of this article used FigDraw (https://www.figdraw.com/#/paint_about). MAPK, mitogen-activated protein kinase; JAK/STAT, Janus kinase/signal transducer and activator of transcription

Mechanical stretching of cardiomyocytes: As the right ventricle undergoes dilation and remodeling, cardiomyocytes are subjected to mechanical stretching and stress, which activates [13] certain signaling pathways [14,15], subsequently activating integrin-mediated signal transduction pathways. This includes the MAPK, JAK/STAT, and calpain-dependent pathways, which play a central role in the myocardial hypertrophic response induced by mechanical stress. The MAPK pathway [16] (involving ERKs, JNKs, and p38 MAPKs) and the JAK/STAT pathway are particularly important in the inflammatory response and apoptosis of cardiomyocytes [17]. The activation of these pathways reveals the complex molecular response of cardiomyocytes to mechanical stress and its impact on cardiac function, leading to the release of Tn from the muscle fibers [18], as illustrated in Figure 2.

The Role of Tn in Prognostic Evaluation of Patients With APE

The prognosis of patients with APE depends on various factors, such as hypoxemia, thrombotic burden, right ventricular function, and comorbidities [19]. Tn, as a sensitive and specific biomarker of myocardial injury, can predict the risk of adverse outcomes like short-term and long-term mortality, and right ventricular dysfunction in patients with APE. Additionally, elevated levels of Tn are associated with an increased risk of short-term and long-term mortality in patients with APE [20-23]. Multiple studies have demonstrated that elevated Tn levels are closely related to adverse prognosis and right ventricular dysfunction in patients with APE [24-28]. Numerous studies have shown that in patients with APE, the elevation of Tn is closely linked to right ventricular dysfunction. For instance, one study utilizing echocardiography revealed that Tn I testing aids in identifying patients with RV dilation, who exhibit significantly more segmental defects in lung scans [29]. Some studies have elucidated the individual parameters of right ventricular dysfunction following APE and evaluated the correlations between these parameters and the extent of pulmonary embolism as well as the recovery within six months [30]. Moreover, studies have also found that cardiac Tn T levels are associated with patient clinical outcomes, with high levels of cardiac Tn T linked to adverse 30-day clinical outcomes [31]. This suggests that Tn can serve as an important indicator for assessing right ventricular function. Compared to other prognostic markers, the predictive value of Tn is similar to or slightly lower than the Simplified Pulmonary Embolism Severity Index (sPESI). However, the combined use of both can enhance the accuracy of prognostic assessment in patients [32]. Therefore, Tn testing should be analyzed in conjunction with other prognostic indicator results to improve the accuracy of predictions

Table 1 provides detailed evidence for the above arguments by demonstrating more correlation studies between Tn levels and prognosis in patients with APE, further underscoring its importance in clinical prognostic evaluation.

**Table 1:** Summary of studies on the relationship between elevated troponin levels and adverse prognostic outcomes and functional impairment in patients with acute pulmonary embolism. hsTnT, high-sensitivity troponin T; BNP, brain natriuretic peptide; NT-proBNP, N-terminal pro-b-type natriuretic peptide

The relationship between Tn elevation and adverse outcomes and prognostic indicators in patients with APE	Title	Content
Risk stratification	Highly Sensitive Troponin T Assay in Normotensive Patients With Acute Pulmonary Embolism	The levels of high-sensitivity troponin T (hsTnT) can be used to assess the risk of non-high-risk PE patients.
	Comparison of Risk Assessment Strategies for Not-High-Risk Pulmonary Embolism	Using age-adjusted cutoff values (≥45 pg/mL for patients aged ≥75 years) can precisely differentiate between low-risk and intermediate-risk patients.
The relationship between short-term adverse outcomes and mortality	Predictive Value of the High-Sensitivity Troponin T Assay and the Simplified Pulmonary Embolism Severity Index in Hemodynamically Stable Patients With Acute Pulmonary Embolism: A Prospective Validation Study. Prognostic Impact of Residual Pulmonary	Troponin elevation independently predicts short-term mortality in acute pulmonary embolism (APE) patients, with higher levels indicating increased death risk. Troponin is also significantly linked to the risk of short-term mortality and adverse events.
	Long-Term Prognostic Value of Residual Pulmonary Vascular Obstruction at Discharge in Patients With Intermediate- to High-Risk Pulmonary Embolism	The role of troponin in predicting short-term mortality in pulmonary embolism patients, but the role in predicting long-term adverse outcomes is unclear.
	Predicting Short-Term Mortality in Patients With Pulmonary Embolism: A Simple Model	The levels of hsTnT are related to 30-day adverse outcomes (death, need for catecholamines, intubation, or cardiopulmonary resuscitation).
	Predictive Value of Biomarkers for the Prognosis of Acute Pulmonary Embolism in Japanese Patients: Results of the Tokyo CCU Network Registry	Troponin, BNP, and blood glucose levels are useful prognostic biomarkers for acute pulmonary embolism in Japanese patients.
	Prognostic Value of Troponins in Acute Nonmassive Pulmonary Embolism: A Meta-Analysis	Elevated troponin levels have prognostic significance in patients with acute non-massive pulmonary embolism, correlating significantly with a heightened risk of short-term mortality, PE-related mortality, and severe adverse events. These levels are indicative of a patient subset at increased risk for short-term mortality and serious adverse events.
	Troponin Dependent 30-Day Mortality in Patients With Acute Pulmonary Embolism	30-day mortality was strongly associated with troponin concentration useful for improving risk stratification, treatment strategies, and outcomes in PE patients.
Long-term adverse outcomes (complicated course)	Can Elevated Troponin I Levels Predict Complicated Clinical Course and Inhospital Mortality in Patients With Acute Pulmonary Embolism?	Elevated levels of cardiac troponin I were found to be associated with the occurrence of a complicated course.
	Highly Sensitive Troponin T Assay in Normotensive Patients With Acute Pulmonary Embolism	Highly sensitive troponin T assays may be capable of improving risk stratification of non-high-risk PE.
Uncertain long-term or short-term	Comparison of Two Methods for Selection of Out of Hospital Treatment in Patients With Acute Pulmonary Embolism	The elevation of hsTnT levels was significantly associated with the mortality and adverse outcomes of patients.
	Implications of Elevated Cardiac Troponin in Patients Presenting With Acute Pulmonary Embolism: An Observational Study	Elevated cTn is an independent predictor of short- and long-term mortality.
	Optimal Follow-Up After Acute Pulmonary Embolism: A Position Paper of the European Society of Cardiology Working Group on Pulmonary Circulation and Right Ventricular Function, in Collaboration With the European Society of Cardiology Working Group on Atherosclerosis and Vascular Biology, Endorsed by the European Respiratory Society	A thorough risk factor evaluation is advised at the three-month checkup, with early intervention for specific risks like smoking and hypertension.
	Clinical Value of Cardiac Color Ultrasound and Cardiac Troponin T Combined With Dynamic Electrocardiogram in Treatment of Acute Pulmonary Embolism	cTnT can also evaluate the prognosis of APE; but the electrocardiogram has little significance in evaluating the prognosis of APE.
integration of imaging parameters	Incremental Prognostic Value of Troponin I and Echocardiography in Patients With Acute Pulmonary Embolism	Elevated cardiac troponin I is crucial for identifying high-risk patients, but a normal echocardiogram and negative troponin I levels best identify those at the lowest risk of early mortality.
	Risk Stratification of Pulmonary Embolism	Combining it with right heart function assessment enables the identification of patients at a heightened risk of early adverse events.
	National Early Warning Score-2 for Identification of Patients with Intermediate-High-Risk Pulmonary Embolism	The addition of troponin testing and echocardiography improved the specificity of NEWS2, although it was not superior to Bova.
The relationship between right ventricular dysfunction	Cardiac Troponin I Elevation in Acute Pulmonary Embolism Is Associated With Right Ventricular Dysfunction	Troponin I testing helps to identify patients with RV dilation, who have significantly more segmental defects in pulmonary scan.
	Predictive Value of the High-Sensitivity Troponin T Assay and the Simplified Pulmonary Embolism Severity Index in Hemodynamically Stable Patients With Acute Pulmonary Embolism: A Prospective Validation Study	Tn elevation was related to the occurrence and degree of right ventricular dysfunction in patients with APE, and the higher the Tn level, the more severe the right ventricular dysfunction.
	Correlation of Heart-Type Fatty Acid–Binding Protein With Mortality and Echocardiographic Data in Patients With Pulmonary Embolism at Intermediate Risk	The higher the Tn level, the more severe the right ventricular dysfunction.
	Comparison of Risk Assessment Strategies for Not-High-Risk Pulmonary Embolism	The elevation of Tn is associated with the occurrence and severity of right ventricular dysfunction in patients with APE.
	Right ventricular dysfunction in acute pulmonary embolism: NT-proBNP vs. troponin T	Individual parameters of right ventricular dysfunction after acute pulmonary embolism were evaluated for their correlation with the degree of pulmonary embolism and the recovery within six months.
	Relationship Between CHA2DS2-VASc Score and Right Ventricular Dysfunction in Patients With Acute Pulmonary Thromboembolism	The CHA2DS2-VASc score is an independent predictor of RVD in patients with acute PTE.
	Prognostic Value of Right Ventricular Dysfunction or Elevated Cardiac Biomarkers in Patients With Low-Risk Pulmonary Embolism: A Systematic Review and Meta-Analysis	In low-risk patients with acute PE, the presence of RV dysfunction on admission was associated with early mortality.
The relationship and interaction of other prognostic indicators	Predictive Value of the High-Sensitivity Troponin T Assay and the Simplified Pulmonary Embolism Severity Index in Hemodynamically Stable Patients With Acute Pulmonary Embolism: A Prospective Validation Study	The prognostic accuracy of hsTnT is on par with or slightly below the simplified Pulmonary Embolism Severity Index (sPESI). Using hsTnT alongside sPESI enhances prognostic predictions for both acute and long-term patient outcomes
	Cardiac Troponin Testing and the Simplified Pulmonary Embolism Severity Index. The SWIss Venous ThromboEmbolism Registry (SWIVTER)	Although troponin testing may not be necessary for patients with low sPESI, it adds prognostic value for early mortality and recurrence in patients with high sPES.
	Comparison of Risk Assessment Strategies for Not-High-Risk Pulmonary Embolism	The study evaluated hsTnT as an alternative to heart-type fatty acid-binding protein (H-FABP) for prognostic purposes, as hsTnT is more commonly available in routine hospital lab.
	Measurement of High-Sensitivity Cardiac Troponin in Pulmonary Embolism: Useful Test or a Clinical	Integrating high-sensitivity cardiac troponin (hs-cTn) with the sPESI model improves risk stratification over either alone. It's yet to be confirmed if combining hs-cTn with other biomarkers betters acute PE patient management.
	Correlation of Heart-Type Fatty Acid-Binding Protein With Mortality and Echocardiographic Data in Patients With Pulmonary Embolism at Intermediate Risk	H-FABP is a significant mortality predictor in intermediate-risk PE patients, correlates better with mortality than troponin I, and may refine management strategies for this challenging patient group.

Current Application of Tn in Clinical Decision Support for Patients With APE

The application of Tn in clinical decision support for patients with APE is of significant importance, aiding physicians in assessing patient prognosis risk and formulating appropriate treatment plans, and follow-up schedules. Presently, numerous global guidelines and consensus statements recommend the use of Tn for clinical decision-making in patients with APE, including the following key instances: 2014 ESC Guidelines on the diagnosis and management of APE [33]. Regarding recommendations on risk stratification for acute non-high-risk pulmonary embolism cases, measurement of cardiac Tn is suggested. For intermediate-risk patients, assessment of right ventricular function using echocardiography or CT, as well as evaluation of myocardial injury using laboratory biomarkers, should be considered for further risk stratification. This further validates their utility as indicators for prognostic evaluation in patients with APE. Continuing on this topic, in 2019, the Pulmonary Embolism Response Team (PERT) Consortium published *Diagnosis, Treatment and Follow Up of APE: Consensus Practice from the PERT Consortium*, pointing out that Tn is one of the major markers for prognosis assessment in patients with APE. Once acute PE is diagnosed, risk stratification using clinical presentation, systolic blood pressure, heart rate, respiratory rate, oxygen requirements, PESI or sPESI, imaging for RV dysfunction (CT or echocardiography), and/or biomarkers (Tn, b-type natriuretic peptide [BNP], or N-terminal pro-b-type natriuretic peptide [NT-proBNP]) is recommended to guide treatment decisions and follow-up frequency.

In 2019, the PERT Consortium published *Diagnosis, Treatment and Follow-Up of Acute Pulmonary Embolism: Consensus Practice from the PERT Consortium* [34]. Also in 2019, the European Society of Cardiology (ESC) and the European Respiratory Society (ERS) released guidelines for the diagnosis and management of APE [4]. It is recommended that the simplified Pulmonary Embolism Severity Index (sPESI) be used primarily as a rule-out criterion rather than for risk stratification in patients with APE, to determine the need for right ventricular function assessment or thrombolysis treatment.

However, the 2019 Guidelines on the Diagnosis and Management of Acute Pulmonary Embolism [35] suggest that right ventricular function should be assessed through imaging (such as transthoracic echocardiography or CTPA) or laboratory biomarkers (mainly troponin and B-type natriuretic peptide) only when clinical risk scores indicate elevated risk. Assessment is not necessary if clinical risk classifications show low or intermediate risks. Subsequently, in 2021, the European Respiratory Society (ERS) published the *Best Follow-Up for Acute Pulmonary Embolism*, recommending that Tn testing be performed on all patients with APE before discharge to assess their long-term prognostic risk and to develop individualized follow-up strategies based on Tn levels and other risk factors [36]. This is an overview of the application of Tn in clinical decision support for patients with APE in various global guidelines and consensus. To illustrate the content and differences of these guidelines and consensus more intuitively, we have created a timeline chart.

**Figure 3:**
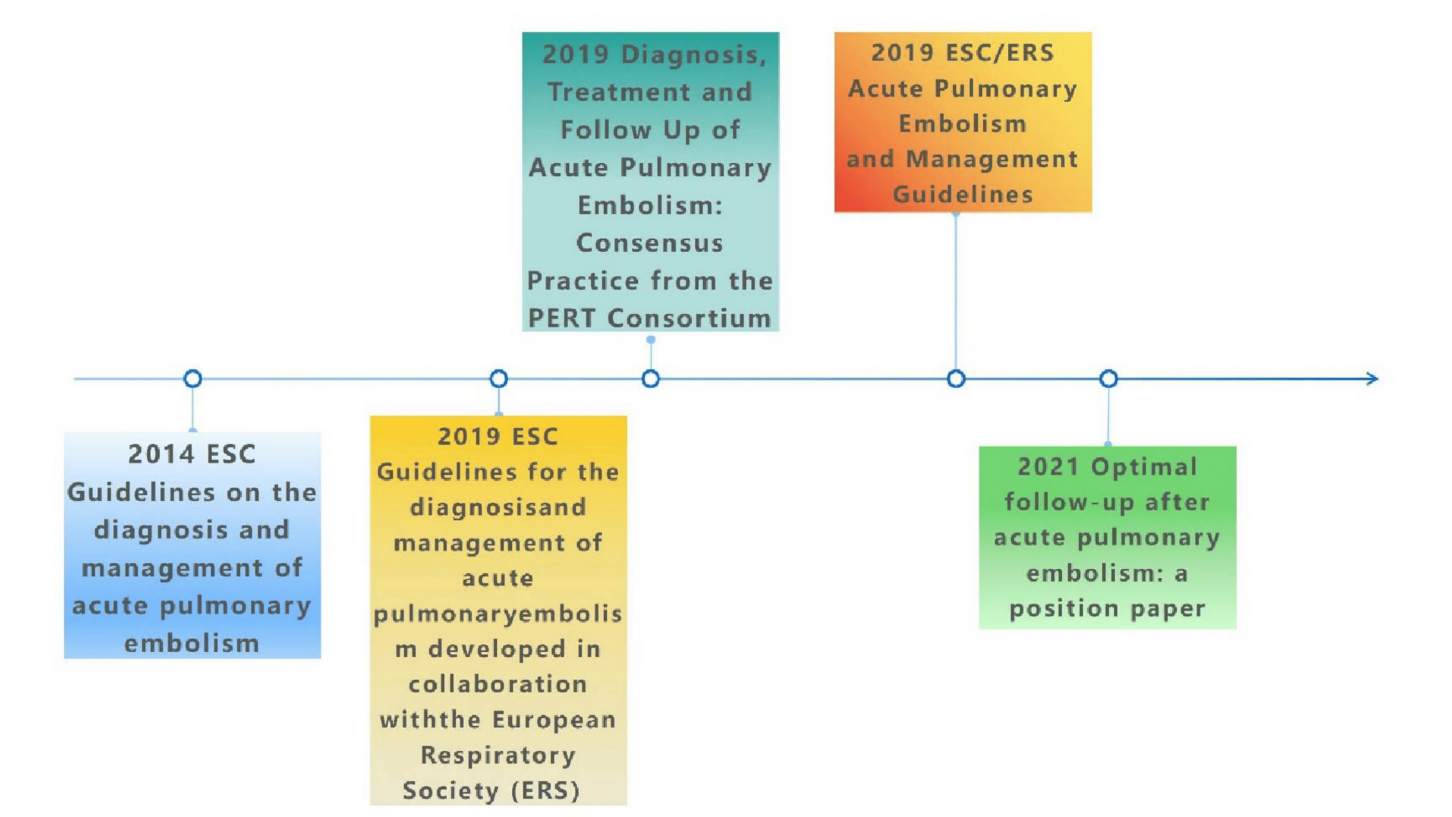
Guidelines and management of acute pulmonary embolism: a cross-sectional comparison from 2014 to 2021. The authors of this article used FigDraw (https://www.figdraw.com/#/paint_about).

At the same time, to visually demonstrate the comparison between different guidelines, Table 2 summarizes their recommendations on the application of Tn testing in APE, methods for assessing risk levels, and guidance for thrombolytic therapy decision-making (Figure 3 for a visual comparison).

**Table 2:** Troponin application, risk assessment, and testing recommendations from multiple guidelines.

Item	Summary of recommendations for the application of troponin	Risk assessment and thrombolytic therapy recommendations	Recommended level of testing
2014 ESC	High-sensitivity cardiac troponin T and cardiac troponin I have predictive value for acute pulmonary embolism (APE).	Can be used to assess risk and decide on thrombolytic therapy	Comprehensive assessment, used in conjunction with other indicators
2019 ERS	Cardiac ultrasound and troponin measurement guide early treatment decisions. High-risk patients do not require immediate troponin testing.	Right ventricular dysfunction and elevated troponin in intermediate-risk patients suggest higher risk	Priority on comprehensive assessment, including clinical and imaging examinations
2019 PERT Consortium	Troponin is an important biomarker for assessing the severity and prognosis of APE.	Patients with elevated troponin may require more aggressive consideration of thrombolytic therapy.	Emphasizing comprehensive assessment, including clinical scoring, imaging, and other biomarkers.
2019 Optimal Follow-Up	Troponin measurement is recommended, with a strong emphasis on comprehensive follow-up and risk factor assessment.	Genetic thrombotic disorder testing supports anticoagulation therapy decisions	Greater emphasis on comprehensive assessment, incorporating various examinations and patient conditions
2019 ESC	High-risk patients are recommended for troponin measurement, while low-risk patients can help exclude myocardial injury.	Used in conjunction with clinical prediction rules to differentiate between intermediate and low-risk pulmonary embolism	Comprehensive assessment takes precedence, combined with clinical scoring and imaging approaches

Discussion

In this review, we systematically explored the multifaceted role of Tn in the diagnosis and treatment of APE, covering its mechanisms of elevation, prognostic evaluation, and clinical decision-making. We recognize Tn as a highly sensitive and specific biomarker of myocardial injury that can reflect the severity of right ventricular dysfunction caused by APE, predict the risk of adverse outcomes such as short-term and long-term mortality in patients with APE, and provide a basis for the treatment choices and follow-up plans for patients with APE. However, we also highlight the complexity and diversity of the mechanisms behind the elevation of Tn in patients with APE, the advantages and limitations of Tn in prognostic assessment, the guidance and practical difficulties of Tn in clinical decision support, and its comparison and combined application with other prognostic indicators and clinical guidelines.

Advantages and Limitations of Tn in Clinical Decision Support for Patients With APE

The use of Tn in clinical decision support for patients with APE presents several advantages: simple and rapid testing methods [37,38]. The detection of Tn involves venous blood sampling followed by analysis using enzyme-linked immunosorbent assay (ELISA) or chemiluminescence methods. This approach is simple and rapid, requiring no complex instrumentation or operations. It can be performed in regular laboratories or emergency departments, with results available within minutes, facilitating timely decision-making by clinicians.

Relatively low cost: The cost of Tn testing is much lower compared to other examinations, such as echocardiography or CT pulmonary angiography. This is particularly advantageous for regions or hospitals with limited resources, as it can save on medical expenses and improve healthcare efficiency.

However, the use of Tn in clinical decision support for patients with APE also has several limitations: lack of uniform standards and thresholds [39]. Currently, there are four common types of Tn used clinically: cardiac Tn I (cTnI), cardiac Tn T (cTnT), high-sensitivity cardiac Tn I (hs-cTnI), and high-sensitivity cardiac Tn T (hs-cTnT).​​​​ These may have different detection methods and results in various laboratories and with different instruments, leading to a lack of unified standards and thresholds [36]. Additionally, different studies might use various criteria for elevated Tn levels to define high-risk or intermediate-risk patients with APE​​​​​​​, resulting in a lack of uniform grading and treatment guidance.

Possible influence by other factors: Tn levels can be affected by other conditions, such as concomitant myocarditis, pericarditis, heart failure, coronary artery atherosclerosis, etc. Therefore, when assessing Tn levels in patients with APE, it is crucial to exclude other conditions that can cause elevated Tn levels. Conditions such as myocarditis, pericarditis, heart failure, and coronary artery atherosclerosis can also lead to elevated Tn levels, which may reduce the reliability of Tn as a single diagnostic indicator. Given these advantages and limitations, clinicians encountering patients suspected of APE need to make comprehensive judgments by combining the patient's clinical symptoms, medical history, and other laboratory test results. For example, in a patient with a history of heart disease presenting with dyspnea, even if Tn​​​​​​​ levels are elevated, the physician needs to consider other possible causes.

As detailed in the Results section, Tn​​​​​​​ levels are an important predictor of prognosis in patients with APE

Table 1 summarizes key findings from multiple studies regarding Tn​​​​​​​ levels. The aggregation of these data points to a series of key considerations about risk assessment and patient management, which we will now analyze and discuss. Specifically, the determination of hsTnT in identifying non-high-risk PE patients suggests its potential value in refined patient management. Although the studies in the table generally support Tn as an independent predictor of adverse prognosis, the data on its role in long-term prognosis are not consistently conclusive [40]. This difference may stem from the heterogeneity of study design, the variation of the patient population, or the length of follow-up time, such as the study by El-Menyar et al. showed that the overall 30-day and three-year mortality rates were 10.0% and 22.3%, respectively [41]. This points out the direction for future research, where we need more standardized study designs to clarify the role of Tn in long-term prognosis. Meanwhile, when interpreting the results of the studies in the table, we must also take into account their limitations. For example, most studies focused on specific patient populations, which may limit the generalizability of these results. In addition, some studies were observational and could not establish causal relationships [42]. For future research, more prospective, randomized controlled trials are needed to validate the role of Tn in different APE patient subgroups. In particular, studies are required on the combined use of Tn with other emerging biomarkers, as well as how to implement these findings in different clinical settings

Furthermore, in the diagnosis and treatment of APE, the combined use of imaging techniques (such as echocardiography) and biomarkers (such as Tn I) is crucial for assessing the prognosis of patients. Echocardiography is a noninvasive examination that can evaluate cardiac structure and function, especially right ventricular function.

Based on the information in the table, when echocardiography shows normal results and cardiac Tn​​​​​​​ I levels are negative, it can predict a low early mortality risk for the patient. This means that if echocardiography does not indicate right ventricular enlargement or dysfunction, and cardiac Tn​​​​​​​ I levels are not elevated, the likelihood of the patient developing severe pulmonary embolism (such as acute right ventricular dysfunction) is relatively low [43].

Conversely, elevated levels of cardiac Tn​​​​​​​ I are closely associated with right ventricular dysfunction, which is particularly important in clinical prognostic assessments. Right ventricular dysfunction is a significant adverse prognostic factor in APE [44]. Testing for cardiac Tn​​​​​​​ I can help physicians identify patients who show signs of right ventricular dilation. These patients are more likely to exhibit more extensive segmental pulmonary vascular obstruction in pulmonary CT scans.

Therefore, the combined use of echocardiography and cardiac Tn I levels can more accurately identify those patients at high risk of pulmonary embolism, providing them with timely and appropriate treatment to improve clinical outcomes. This integrated assessment approach helps clinicians decide whether further therapeutic interventions are needed, such as thrombolytic therapy or intensified anticoagulation treatment, as well as whether closer monitoring and follow-up are necessary. Through this method, physicians can more accurately stratify patients' treatment and monitoring strategies, especially in managing high-risk patients during the acute phase.

The similarities and differences of Tn​​​​​​​ application in the pulmonary embolism section of global guidelines and consensus

In the Results section of this review, we presented the recommendations of five major guidelines and expert consensus on the application of Tn​​​​​​​ in the diagnosis and treatment of APE (Table 2). Now, by analyzing the similarities and differences between these guidelines and their potential impact on clinical practice, it is not difficult to find that regarding the recommendation level of Tn​​​​​​​ testing: All the guidelines emphasize the importance of troponin testing, but also point out that it cannot be used alone; it should be combined with other indicators and examinations for a comprehensive assessment.​​​​​​​ Among them, 2014 ESC, 2019 ERS, 2019 PERT Consortium, and 2019 ESC all recommend Tn​​​​​​​ testing for all suspected or confirmed patients with APE​​​​​​​, especially noteworthy is that 2019 ERS gave some key and specific values of Tn​​​​​​​, such as the proportion of Tn​​​​​​​ elevation in patients with APE​​​​​​​: about 30% in patients using conventional testing methods, and about 60% in patients using high-sensitivity testing methods had elevated Tn​​​​​​​ I or T [20]. Also, regarding the negative predictive value of high-sensitivity Tn​​​​​​​ testing: for example, in a prospective multicenter cohort study of 526 hemodynamically stable patients, the negative predictive value of high-sensitivity Tn​​​​​​​ T concentration for excluding adverse in-hospital clinical outcomes was 98% when it was lower than 14 pg/mL. Adjusted high-sensitivity Tn​​​​​​​ T cutoff values for different age groups (< 75-year-old patients as ⩾14 pg/mL, ⩾75-year-old patients as ⩾45 pg/mL) may further improve the negative predictive value of this biomarker [45]. The 2019 Optimal Follow-Up After Acute PE: ESC Position Paper emphasizes the importance of systematic cardiovascular risk assessment for all PE patients, including the use of biomarkers such as troponin​​​​​​​. This may be because the recommendation to use myocardial injury biomarkers in high-risk cases is likely based on their sensitivity and specificity to the severity of APE. The levels of myocardial injury biomarkers may be directly proportional to the severity of the disease, aiding in distinguishing between high-risk and low-risk cases. On the other hand, some guidelines may not recommend the use of myocardial injury biomarkers in all situations as the primary diagnostic tool. This could be because, in certain cases, other diagnostic methods (such as imaging studies) may provide more direct or specific information. The differences between the guidelines may reflect recent advancements in research on the diagnosis and treatment methods for APE. For instance, with the advancement of medical imaging technologies, some guidelines may lean more towards using these technologies for patient assessment. Regarding recommendations for thrombolytic therapy, the 2014 ESC, 2019 ERS, 2019 PERT Consortium, and 2019 ESC, all suggest conducting right ventricular function and cardiac Tn​​​​​​​ tests for intermediate-risk patients. If right ventricular dysfunction and elevated Tn​​​​​​​ levels are found, the patient is classified as high-risk, and thrombolytic therapy may be considered, unless contraindicated. On the other hand, the 2019 Optimal Follow-up after acute PE: ESC position paper recommends testing for hereditary thrombophilic disorders in high-risk patients to support decisions regarding anticoagulant therapy.

Future research and practice directions

Future research should focus on standardizing the measurement and application of Tn and explore its combined use with emerging biomarkers and diagnostic technologies to accurately determine the severity of APE and identify the best treatment strategies.

## Conclusions

In summary, Tn plays a vital role in the diagnosis, treatment, and prognosis of APE. Tn elevation usually reflects the overload, ischemia, and possible necrosis of the right ventricle due to pulmonary artery obstruction, which not only provides biomarker evidence for APE but also is a key factor for prognosis assessment. This article reveals the multiple roles of Tn in risk stratification and treatment strategy formulation of patients with APE by analyzing many studies, including the key data from 2019 ERS. Therefore, in APE management, combining Tn testing and comprehensive cardiovascular risk assessment will provide clinicians with a more accurate prognosis assessment and treatment decision basis. However, Tn level elevation may also be affected by other cardiac lesions or noncardiac factors, so the overall clinical situation of the patient needs to be considered comprehensively in clinical application. In addition, different types of Tn testing may have standardization and consistency issues among different laboratories, and further studies are needed to determine their role in long-term prognosis assessment.
